# Fuzzy Number Addition with the Application of Horizontal Membership Functions

**DOI:** 10.1155/2015/367214

**Published:** 2015-06-23

**Authors:** Andrzej Piegat, Marcin Pluciński

**Affiliations:** Faculty of Computer Science and Information Systems, West Pomeranian University of Technology, Żołnierska 49, 71-210 Szczecin, Poland

## Abstract

The paper presents addition of fuzzy numbers realised with the application of the multidimensional RDM arithmetic and horizontal membership functions (MFs). Fuzzy arithmetic (FA) is a very difficult task because operations should be performed here on multidimensional information granules. Instead, a lot of FA methods use *α*-cuts in connection with 1-dimensional classical interval arithmetic that operates not on multidimensional granules but on 1-dimensional intervals. Such approach causes difficulties in calculations and is a reason for arithmetical paradoxes. The multidimensional approach allows for removing drawbacks and weaknesses of FA. It is possible thanks to the application of horizontal membership functions which considerably facilitate calculations because now uncertain values can be inserted directly into equations without using the extension principle. The paper shows how the addition operation can be realised on independent fuzzy numbers and on partly or fully dependent fuzzy numbers with taking into account the order relation and how to solve equations, which can be a difficult task for 1-dimensional FAs.

## 1. Introduction

Fuzzy arithmetic [1–14] is used in uncertainty theory [15–20], grey systems [21, 22], granular computing [9, 23], computing with words [23–26], decision-making [27, 28], and other sciences and engineering branches [4, 26, 29, 30]. Authors of first concepts of the fuzzy arithmetic (shortly FA) based on *L*-*R* (left-right) fuzzy numbers were Dubois and Prade [1]. With years, FA has been improved and its new versions have been introduced, for example, the popular *α*-cuts' version [4, 6, 31], which in this paper will be called *μ*-cuts' FA. In general, all versions of FA can be divided [4] into elementary FA, standard FA, and advanced FA versions. Examples of advanced FA methods can be the generalized vertex method [32], constrained FA [7, 8, 21], algorithmic FA [9], transformation method and extended transformation method [4], and inverse FA [4]. It seems that mostly used FA versions are based on *α*-cuts [2] and on Moore's interval arithmetic (shortly IA) [33–35]. In the FA literature, many examples of practical FA applications can be found, for example, [4, 26, 27, 36, 37]. Practical problems require effective FAs which would enable solving uncertain linear and nonlinear equation systems, differential equations, integral calculations, and so forth [38, 39]. Therefore, many scientists investigate these problems and publish achieved results from this area [40–64]. However, almost always they emphasise that the achieved level of FA is not satisfactory and that further investigations are necessary. Therefore, investigations on IA and FA have been continued nonstop. It is testified by new publications in journals, conferences, and new books. Though FA has achieved many application successes, it further on has many weak points. For example, Dymova and Sevastjanov report in their papers [27, 61, 62] the present FA has considerable difficulties in solving even simple equations with one unknown variable. There exist also difficulties in defining neutral and inverse elements for addition and multiplication.

The most popular version of FA is the arithmetic based on *α*-cuts, where an *α*-cut of a fuzzy set *A*, denoted as *A*_*α*_, is defined by (1), where *X* means domain of the set and *x* means an element of this domain [4]. Consider

(1) $$\begin{array}{c} A_{\alpha }=cut_{\alpha }\left(\;\tilde{A}\;\right)=\left\{\;x\in X\;|\;\mu_{\tilde{A}}\left(\;x\;\right)\geq \alpha \;\right\}. \end{array}$$

FA based on *α*-cuts uses principles of the classical Moore's interval arithmetic [33, 34] for realisation of elementary arithmetic operations such as addition, subtraction, multiplication, and division. Though this arithmetic has many applications, its possibilities are limited, because of some drawbacks, which are commented on also by Moore himself in his books. For example, if *X*, *Y*, and *Z* are intervals, the distributive law,

(2) $$\begin{array}{c} X\cdot \left(\;Y+Z\;\right)=X\cdot Y+X\cdot Z, \end{array}$$

does not always hold. Additionally, an additive inverse of an interval is not defined. If $X=[\underline{x},\bar{x}]$ , then $X+(-X)=[\underline{x}-\bar{x},\bar{x}-\underline{x}]\neq [0,0]$ .

As Dymowa shows in [27], there are great difficulties in solving even simple interval equations with one unknown. Let us assume that in a system there exists the dependence *a* + *x* = *c*. This dependence can be presented in few equivalent forms:

(3) a+x=c,a=c−x,x=c−a,a+x−c=0.

If only approximate values of *a* and *c* are known, *a* ∈ [1,3] and *c* ∈ [2,5], and value *x* is not known, then, on the basis of (3), four interval equations (4)–(7) can be written:

(4) 1,3+x_,x¯=2,5,

(5) 1,3=2,5−x_,x¯,

(6) x_,x¯=2,5−1,3,

(7) 1,3+x_,x¯−2,5=0.

Solving (4), two equations, $1+\underline{x}=2$ and $3+\bar{x}=5$ , are obtained. They give the solution $\left[\underline{x},\bar{x}]=[1,2\right]$ . Solving (5), two equations, $1=2-\bar{x}$ and $3=5-\underline{x}$ , are obtained. They give the solution $\left[\underline{x},\bar{x}]=[2,1\right]$ being an inverse interval. Solving (6), the solution [−1,4] is obtained and from (7) we get the solution [4, −1], which is also an inverse interval. Thus, various solutions, [1,2], [2,1], [−1,4], and [4,1], are obtained depending on extension form of the original dependence. In the case of more complicated mathematical dependencies, the solution number can be considerably higher.

Another paradox of FA based on *μ*-cuts and Moore arithmetic (shortly *μ*-cuts' FA) [33–35] is not satisfying the cancellation law for multiplication. For example, equation *XZ* = *YZ* in the general case does not mean that *X* = *Z*. It can be testified by an example shown below in which notation [1,2, 3] means the triangle membership function (shortly MF) with support beginning, core position, and support end. Consider

(8) XZ1,2,3·−1,0,1=−3,0,3=YZ=2,2.5,3·−1,0,1=−3,0,3,

but

(9) $$\begin{array}{c} X=\left[\;1,2,3\;\right]\neq Y=\left[\;2,2.5,3\;\right]. \end{array}$$

Because of this feature of *μ*-cuts' FA, transformations of formulas are not allowable. Why? It will be shown further on. Next important paradox of *μ*-cuts' FA is the observation that, during calculation of results of nonlinear formulas, for example, *C* = *A* − *A* ^2^, we obtain different, nonunique solutions depending on which form of the formula is used: *C*_1_ = *A* − *A* ^2^,  *C*_2_ = *A*(1 − *A*), or *C*_3_ = (*A* − 1)+(1 − *A*)(1 + *A*). For *A* = [0,1, 2], three different solutions are obtained: *C*_1_ = [−4,0, 2],  *C*_2_ = [−2,0, 2], and *C*_3_ = [−4,0, 4]; see Figure 4. Which solution is correct? The above phenomenon means that each transformation of an equation form, in the case of *μ*-cuts' FA, can change its solution and that solutions are not unique. Further on, it will be shown that in the case of the multidimensional RDM FA such paradoxes do not occur.

The above examples show that the classical IA, which is a basis of the *μ*-cut version of FA, is not ideal, though it can solve certain problems. Therefore, it can and should be further developed. Further on, a version of FA that is based on horizontal membership functions (MFs) and on *μ*-cuts will be presented. It also applies the multidimensional RDM arithmetic (M-RDM arithmetic) which has been elaborated by Andrzej Piegat. It has been developed together with coworkers: Marek Landowski, Marcin Pluciński, and Karina Tomaszewska [10, 37, 65–70].

## 2. Horizontal Membership Function

**Figure 1:**
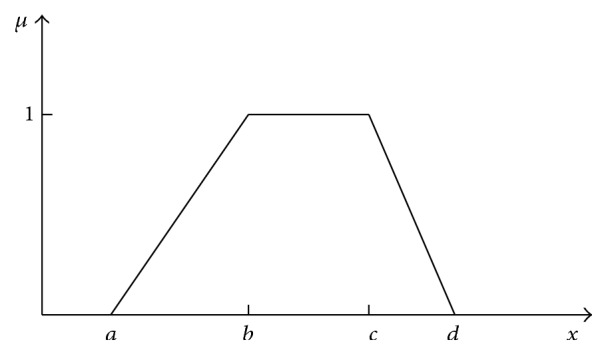
Trapezium membership function.

Fuzzy systems use vertical membership functions which were introduced by Zadeh [23]. They have the following form: *μ* = *f*(*x*), where *x* is an independent variable and *μ* is a dependent one.  Figure 1 shows an example of the trapezoidal MF and formula (10) gives its description:

(10) xa,b:  μx=x−ab−a,xb,c:  μx=1,xc,d:  μx=d−xd−c.

**Figure 2:**
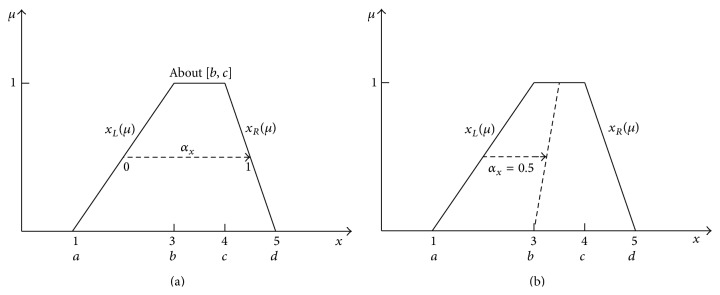
Visualization of the horizontal approach to fuzzy membership functions.

Vertical MF realises the mapping *x* → *μ*. The present fuzzy arithmetic is based on just such MFs. The idea of horizontal MFs has been elaborated by Andrzej Piegat. In this paper, an example of a trapezium MF will be presented but horizontal MFs can be used for all types of MFs. A function *μ*(*x*) is unambiguous in the direction of the variable *μ* (Figure 1) and ambiguous in the direction of *x*. Therefore, it seems impossible to define a membership function in the *x*-direction. The function from Figure 1 assigns two values of *x*, *x*_*L*_(*μ*) and *x*_*R*_(*μ*), for one value of *μ*. However, let us introduce the RDM variable: *α*_*x*_ ∈ [0,1]. This variable has meaning of the relative-distance-measure and allows for determining of any point between two borders *x*_*L*_(*μ*) and *x*_*R*_(*μ*) of the function (Figure 2). RDM variable *α*_*x*_ takes a value of zero on the left border and a value of 1 on the right border of the function. Between the left border and the right border it takes fractional values. The idea of RDM variables was successfully used in the multidimensional IA [37, 67–70]. The multidimensional IA has shown that full and precise solutions of granular problems have form of multidimensional granules that cannot be explained and understood in terms of 1-dimensional approaches.

Formula (11) defines the left border and the right border of the trapezium MF (Figure 2). Consider

(11) xL=a+b−aμ,xR=d−d−cμ.

RDM variable *α*_*x*_ allows for a gradual transition of points between the left border and the right border. The interval *x*(*μ*) in Figure 2 is defined by the following formula:

(12) x=xL+xR−xLαx,αx∈0,1,x=a+b−aμ+d−a−μb−a+d−cαx.

**Figure 3:**
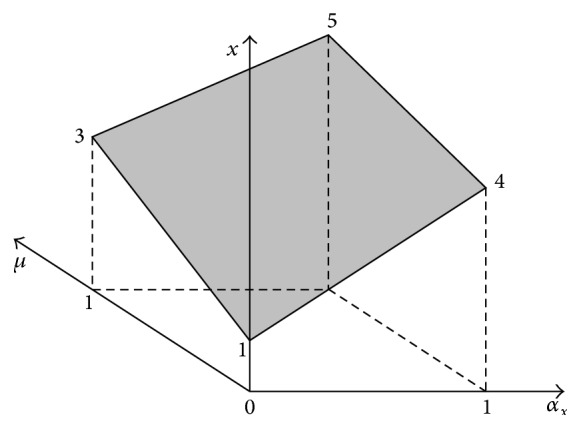
The horizontal membership function *x* = (1 + 2*μ*)+(4 − 3*μ*)*α*_*x*_, where *α*_*x*_, *μ* ∈ [0,1], corresponding to the function from Figure 2, in the 3D space as the unambiguous function.

It can be noted in formula (12) that *x* = *f*(*μ*, *α*_*x*_) is an unambiguous function existing in the 3D space, which can be seen in Figure 3.

It should be also noticed that RDM variables *α*_*i*_ introduce the continuous Cartesian coordinate system in interval and fuzzy arithmetic calculations. In Moore's arithmetic and in *μ*-cuts' FA based on it, only borders of intervals or *μ*-cuts, for example, [*a*, *b*]+[*c*, *d*] = [*a* + *b*, *c* + *d*], take part in calculations. Insides of intervals are not taken into account. This fact hinders solving of more complicated problems and results in many paradoxes observed in IA and FA calculations. It also deprives IA and FA of many mathematical properties which the conventional mathematics of crisp numbers has. Few of these properties will be presented further on.

Thanks to the fact that RDM variables *α*_*i*_ introduce in interval calculations local and continuous Cartesian coordinate system, RDM FA possesses almost the same mathematical properties as arithmetic of crisp numbers.

### 2.1. Additive Inverse Element −*X* of Element *X*

In *μ*-cuts' FA, trapezoidal membership function can be defined as a fuzzy interval with left border *x*_*L*_(*μ*) = *a* + (*b* − *a*)*μ* and right border *x*_*R*_(*μ*) = *d* − (*d* − *c*)*μ* (formula (13) and Figure 2). Consider

(13) $$\begin{array}{c} X\left(\;\mu \;\right)=\left[\;x_{L}\left(\;\mu \;\right),x_{R}\left(\;\mu \;\right)\;\right]. \end{array}$$

A negative element −*X* is determined by the following formula:

(14) $$\begin{array}{c} -X\left(\;\mu \;\right)=-\left[\;x_{L}\left(\;\mu \;\right),x_{R}\left(\;\mu \;\right)\;\right]=\left[\;-x_{R}\left(\;\mu \;\right),-x_{L}\left(\;\mu \;\right)\;\right]. \end{array}$$

The subtraction result *X*(*μ*) − *X*(*μ*) determined by (15) is a fuzzy interval of nonzero span which means that, in *μ*-cuts' FA additive, inverse element does not exist. Consider

(15) Xμ−Xμ=−xRμ−xLμ,xRμ−xLμ≠0,0.

In the case of RDM FA, the element *X*(*μ*, *α*_*x*_) is determined by (16) and its negative element −*X*(*μ*, *α*_*x*_) by (17). Consider

(16) Xμ,αx=xμ,αx:xμ,αx=xLμ+αxxRμ−xLμ,  αx∈0,1,

(17) −Xμ,αx=xμ,αx:xμ,αx=−xLμ−αxxRμ−xLμ,  αx∈0,1.

It is easy to check that the subtraction result *X* − *X* is exactly equal to zero:

(18) $$\begin{array}{c} X\left(\;\mu ,\alpha_{x}\;\right)-X\left(\;\mu ,\alpha_{x}\;\right)=0. \end{array}$$

Thus, in the case of RDM FA, there exists the inverse additive element −*X*(*μ*, *α*_*x*_).

### 2.2. Distributive Law of Multiplication

In the *μ*-cuts' FA, similarly as in Moore's interval arithmetic, the distributive law holds only in the limited form:

(19) $$\begin{array}{c} X\left(\;\mu \;\right)\left[\;Y\left(\;\mu \;\right)+Z\left(\;\nu \;\right)\;\right]\subseteq X\left(\;\mu \;\right)Y\left(\;\mu \;\right)+X\left(\;\mu \;\right)Z\left(\;\mu \;\right). \end{array}$$

In the multidimensional, continuous RDM FA, the distributive law holds fully. Consider

(20) Xμ,αxYμ,αy+Zμ,αz=Xμ,αxYμ,αy+Xμ,αxZμ,αz,αx,αy,αz∈0,1.

**Proof**

For any three intervals expressed in RDM notation, with *α*_*x*_, *α*_*y*_, *α*_*z*_ ∈ [0,1],

(21) Xμ,αx=xLμ,αx,xRμ,αx=xμ,αx:xμ,αx=xLμ+αxxRμ−xLμ,Yμ,αy=yLμ,αy,yRμ,αy=yμ,αy:yμ,αy=yLμ+αyyRμ−yLμ,Zμ,αz=zLμ,αz,zRμ,αz=zμ,αz:zμ,αz=zLμ+αzzRμ−zLμ,

we have

(22) Xμ,αxYμ,αy+Zμ,αz=xLμ+αxxRμ−xLμ·yLμ+αyyRμ−yLμ+zLμ+αzzRμ−zLμ=xLμ+αxxRμ−xLμyLμ+αyyRμ−yLμ+xLμ+αxxRμ−xLμzLμ+αzzRμ−zLμ=Xμ,αxYμ,αy+Xμ,αxZμ,αy.

### 2.3. Different Forms of Nonlinear Formulas

**Figure 4:**
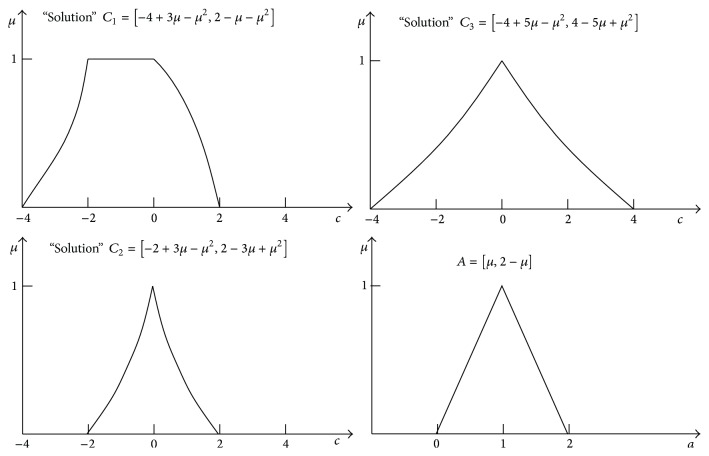
Three different membership functions of “results” of the fuzzy formula *C* = *A* − *A* ^2^ written in three equivalent forms *C*_1_ = *A* − *A* ^2^,  *C*_2_ = *A*(1 − *A*),  *C*_3_ = (*A* − 1)+(1 − *A*)(1 + *A*) obtained with *μ*-cuts' fuzzy arithmetic, where *A* = [*μ*, 2 − *μ*].

Let us consider results of *μ*-cuts' FA and RDM FA for the nonlinear formula *C* = *A* − *A* ^2^, where *A* is the fuzzy interval *A* = [0,1, 2] (Figure 4). This formula can take at least 3 forms: *C*_1_ = *A* − *A* ^2^, *C*_2_ = *A*(1 − *A*), and *C*_3_ = (*A* − 1)+(1 − *A*)(1 + *A*). Fuzzy interval *A* has the form *A* = [*a*_*L*_(*μ*), *a*_*R*_(*μ*)] = [*μ*, 2 − *μ*]. Calculating value of *C* for particular forms, *C*_1_, *C*_2_, and *C*_3_, with the application of *μ*-cuts' FA, three different results are obtained:

(23) C1=−4+3μ−μ2,2−μ−μ2,with  support  span  −4,2,C2=−2+3μ−μ2,2−3μ+μ2,with  support  span  −2,2,C3=−4+5μ−μ2,4−5μ+μ2,with  support  span  −4,4.

Each of the results, *C*_1_, *C*_2_, and *C*_3_, is different. For *μ* = 0, results mean spans (widths) of MF supports. They are equal to [−4,2], [−2,2], and [−4,4]. The differences are considerable. Thus, the formula *C* = *A* − *A* ^2^ cannot be calculated uniquely with *μ*-cuts' FA. If we use the multidimensional RDM FA, then interval *A* is expressed by (24) and its square *A* ^2^ by (25), where *a* is generic variable of *A*. One should notice that functions expressing *A* and *A* ^2^ are fully coupled (correlated); that is, values of *a* and *α*_*a*_ have to be identical both in *A* and in *A* ^2^. Consider

(24) A:  aaL+αaaR−aL=μ+2αa1−μ,

(25) A2:  a2μ+2αa1−μ2,αa∈0,1.

Formula *C*_1_ = *A* − *A* ^2^ can be determined by

(26) C1A−A2:a−a2=μ+2αa1−μ−μ+2αa1−μ2,αa∈0,1.

Formula *C*_2_ = *A*(1 − *A*) can be determined by

(27) C2A1−A:a1−a=μ+2αa1−μ·1−μ+2αa1−μ=μ+2αa1−μ−μ+2αa1−μ2=a−a2,αa∈0,1.

As can be seen, result for the second form *C*_2_ is the same as for *C*_1_. Result for the third form *C*_3_ can be determined by

(28) C3A−1+1−A1+A:a−1+a−11+a=μ+2αa1−μ−1+1−μ+2αa1−μ·1−μ+2αa1−μ=μ+2αa1−μ−1+1−μ+2αa1−μ2=μ+2αa1−μ−μ+2αa1−μ2=a−a2,αa∈0,1.

The result *C*_3_ is identical to *C*_1_ and *C*_2_. As the analysed example shows the multidimensional RDM FA provides unique solutions for nonlinear formulas independently of their mathematical form, it cannot be said about *μ*-cuts' FA. To better realise this fact, particular solutions will be visualised in Figure 4.

One can easily see that all three solutions, *C*_1_, *C*_2_, and *C*_3_, are incorrect. According to the solution *C*_1_, it is possible that the difference *c* = *a* − *a* ^2^ for *a* ∈ [0,2] could be equal to, for example, −4 or +2. However, these values cannot be obtained with any value of *a* ∈ [0,2]. According to the “result” *C*_2_, possible values of *c* = *a* − *a* ^2^ could be equal to +1 or +2. But, in fact, these values are impossible. According to the “result” *C*_3_, possible values of *c* are equal to −4 or +4. However, it is not true.

The full and precise 3D solution, in which formula (obtained with use of the RDM FA) is given by

**Figure 5:**
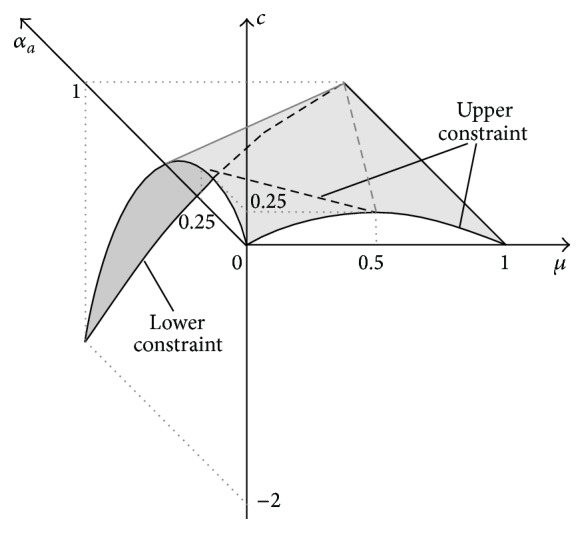
Full and complete 3D result of formula *C* = *A* − *A* ^2^, where *A* = [*μ*, 2 − *μ*], obtained with use of the multidimensional RDM FA (29), with marked upper (right) and lower (left) constraint of 2D membership function.

(29) C:  c=μ−μ2+2αa1−μ−4αa1−μ−4αa21−μ2,

is shown in Figure 5.

If we are interested in the simplified, traditional MF of the full 3D result in the horizontal version, it can be easily determined analytically on the basis of formula (30) for particular membership levels (*μ*-cuts). Consider

(30) CA−A2=cLμ,cRμ=minαa⁡cμ,αa,maxαa⁡cμ,αa,μ,αa∈0,1,

where *c*(*μ*, *α*_*a*_) is determined by formula (29). Extremes of *c*(*μ*, *α*_*a*_) can be detected by usual, analytical function examination. However, one should remember that function extremes can lie not only on domain borders of variable *α*_*a*_ (*α*_*a*_ = 0, *α*_*a*_ = 1) but also inside of the domain (fractional values of *α*_*a*_). Then, the extremes can be detected by identification of critical points of the derivative d*c*(*μ*, *α*_*a*_)/d*α*_*a*_ = 0.

In the analysed problem, examination of the function *c*(*μ*, *α*_*a*_) has shown that its left border *c*_*L*_(*μ*) and right border *c*_*R*_(*μ*) have mathematical form expressed by the following formula:

(31) CA−A2=cLμ,cRμ,cLcμ,αa=1=−2+3μ−μ2,cR0.25for  μ∈0,0.5cμ,αa=0=μ−μ2for  μ∈0.5,1.

**Figure 6:**
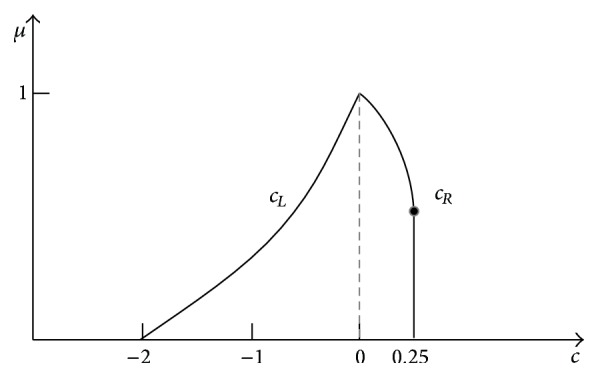
Membership function representing the span of the full 3D result of the fuzzy formula *C* = *A* − *A* ^2^.

Membership function representing the span (width) of the full result *c*(*μ*, *α*_*a*_) of the fuzzy formula *C* = *A* − *A* ^2^ is shown in Figure 6.

Comparison of MFs of results obtained with use of the *μ*-cuts' FA (Figure 4) and with use of the multidimensional RDM FA (Figure 6) shows that “results” provided by the popular *μ*-cuts' FA are excessively broad and false, because they suggest that certain values of the result *c* = *a* − *a* ^2^ are possible (e.g., −4, +2, +4, and others), whereas they in fact are impossible. It can be easily checked analytically or numerically for various allowed values of *a* ∈ [0,2].

Similarly, as the fuzzy formula *C* = *A* − *A* ^2^ has been solved with use of the multidimensional RDM FA, other linear and nonlinear formulas or equations can be solved also. Their solutions usually will be less uncertain than solutions obtained with not-multidimensional methods.

## 3. Addition of Two Independent Triangle Fuzzy Numbers

**Figure 7:**
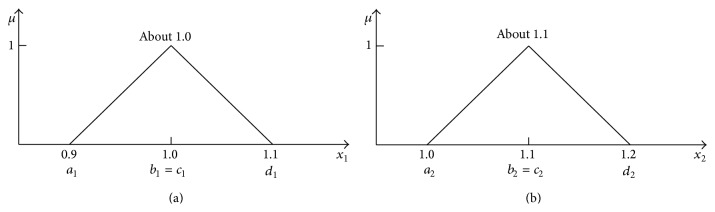
Membership functions of uncertain weights *X*_1_ and *X*_2_ of two stones S1 and S2.

Triangle numbers (Figure 7) are used in practice very frequently. Two numbers “about 1.0” and “about 1.1” can represent weights of two stones, S1 and S2, that were weighed on the scales. The scales have shown *x*_1_ = 1.0 kg for stone 1 and *x*_2_ = 1.1 kg for stone 2. Because the scales have the maximal error ±0.1 kg, stone weights are uncertain and can be described by fuzzy numbers: *X*_1_ is [0.9, 1.0, 1.1] and *X*_2_ is [1.0, 1.1, 1.2].

Horizontal MF of the stone S1 is given by (32) and horizontal MF of the stone S2 is given by (33). Consider

(32) x1=a1+μb1−a1+αx1d1−a11−μ,x1=0.9+0.1μ+0.2αx11−μ,αx1∈0,1,

(33) x2=a2+μb2−a2+αx2d2−a21−μ,x2=1.0+0.1μ+0.2αx21−μ,αx2∈0,1.

If we want to calculate the sum *y* of weights of both stones, then the calculation with horizontal MFs can be made similarly as in a classical arithmetic, without use of the extension principle of Zadeh [37]. It is necessary only when vertical MFs are used. According to this principle, the membership function of the addition result is determined by (34), where *A*_1_ and *A*_2_ are fuzzy numbers [4, 10]. Consider

(34) μA1+A2y=⋁y=x1+x2μA1x1∧μA2x2,∀x1,x2,y∈R.

If horizontal MFs are used, then the sum *y* = *x*_1_ + *x*_2_ is created directly by adding *x*_1_ and *x*_2_ determined by (32) and (33). Consider

(35) x1a1,d1,x2a2,d2,yx1+x2=a1+a2+μb1+b2−a1−a2+αx1d1−a1+αx2d2−a21−μ,y1.9+0.2μ+0.2αx1+αx21−μ,αx1,αx2,μ∈0,1.

**Figure 8:**
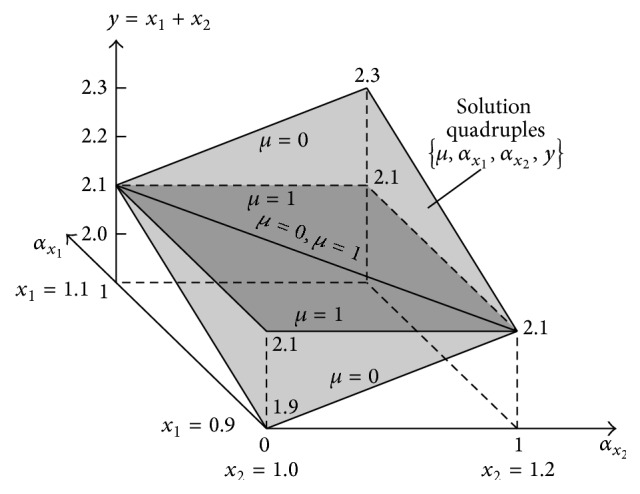
Multidimensional granule of the addition result of two fuzzy numbers “about 1.0” and “about 1.1” being the set of quadruples {*μ*, *α*_*x*_1__, *α*_*x*_2__, *y*} determined by formula (35) and projected onto the 3D space *α*_*x*_1__ × *α*_*x*_2__ × *Y*.

Formula (35) shows that the sum *y* = *x*_1_ + *x*_2_ is not 1-dimensional. It is a function defined in the 4D space because *y* = *f*(*μ*, *α*_*x*_1__, *α*_*x*_2__). Thus, it cannot be visualised but it can be shown as the projection onto the 3D space: *α*_*x*_1__ × *α*_*x*_2__ × *Y* (Figure 8). The fourth dimension *μ* is shown in a form of *μ*-cuts. For *μ* = 0, sum (35) takes a form: *y* = 1.9 + 0.2(*α*_*x*_1__ + *α*_*x*_2__). For *μ* = 1, the sum has value *y* = 2.1 independently of values of *α*_*x*_1__ and *α*_*x*_2__. Surfaces of both *μ*-cuts can be seen in Figure 8.

**Figure 9:**
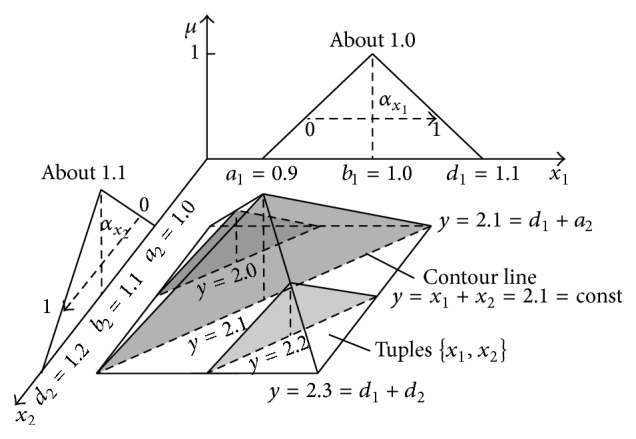
Projection of the full 4D addition result granule onto the 3D space *X*_1_ × *X*_2_ × *μ* with slant contour lines corresponding to particular addition results *y* = *x*_1_ + *x*_2_ = const.

The multidimensional result granule (Figure 8) contains an infinitive number of *μ*-cuts corresponding to particular fractional values of *μ* ∈ [0,1]. Figure 8 shows only two border cuts for *μ* = 0 and *μ* = 1. Apart from the 3D projection shown in Figure 8, other projections of the full 4D granule are also possible.  Figure 9 shows the projection onto the 3D space *X*_1_ × *X*_2_ × *μ*. In this figure, addition results *y* = *x*_1_ + *x*_2_ are shown in a form of contour lines *y* = *x*_1_ + *x*_2_ = const.

Each of the contour lines *y* = const is the set of infinite number of tuples {*x*_1_, *x*_2_} satisfying the condition *x*_1_ + *x*_2_ = *y*. As can be seen in Figure 9, sum *y* = 2.3 can occur only for *x*_1_ = 1.1 and *x*_2_ = 1.2. Instead, sum *y* = 2.1 has considerably greater possibility of occurrence because the number of tuples {(*x*_1_, *x*_2_)∣*x*_1_ + *x*_2_ = *y* = 2.1} is infinitely large. The value *y* = 2.1 occurs, for example, for *x*_1_ = 1.0 and *x*_2_ = 1.1 and for *x*_1_ = 1.01 and *x*_2_ = 1.09. A cardinality of particular sets *Y*_*y*_ [69] can be calculated with the following formula:

(36) Abs  CardyCardy=∫Cmin⁡μx1,μx2ds,

where *C* is a contour line defined as

(37) C=x1,x2 ∣ x1+x2=y,  x1∈a1,d1,  x2∈a2,d2.

The cardinality of particular sets *Y*_*y*_ is equal to an area of *y*-cuts of the granule (Figure 9). It is easy to calculate. For example, the cardinality of the set *Y*_2.1_ equals

(38) Cardy=2.112d2−a22+d1−a12≈0.14142.

It is the greatest of all cardinalities (Max Abs Card(*y*)). It can be shown that the cardinality of particular sets is a square function of the sum *y* = *x*_1_ + *x*_2_ ((39) and (40)).

For *y* < *a*_1_ + *d*_2_,

(39) Abs  CardyCardy=y−a1+a2a2+d1−a1+a22·Max  Abs  Cardy.

For *y* ≥ *a*_1_ + *d*_2_,

(40) Abs  CardyCardy=d1+d2−yd1+d2−d1+a22·Max  Abs  Cardy.

The absolute cardinality of the sum *y* = *x*_1_ + *x*_2_ can take various numeric values. Therefore, it should be normalised to interval [0,1] for convenience ((41) and (42)). The normalised cardinality Norm  Card(*y*) can also be called the relative cardinality Rel Card(*y*).

For *y* < *a*_1_ + *d*_2_,

(41) Norm  CardyRel  Cardy=Abs  CardyMax  Abs  Cardy=y−a1+a2d1−a12.

For *y* ≥ *a*_1_ + *d*_2_,

(42) Norm  CardyRel  Cardy=Abs  CardyMax  Abs  Cardy=d1+d2−yd2−a22.

It should be noticed that formulas (41) and (42) concern fuzzy numbers with an identical support. In the considered example, *d*_1_ − *a*_1_ = *d*_2_ − *a*_2_ = 0.2, so they take the following form:

(43) For  ya1+d2:  Norm  Cardy=y−1.90.22,For  ya1+d2:  Norm  Cardy=2.3−y0.22.

**Figure 10:**
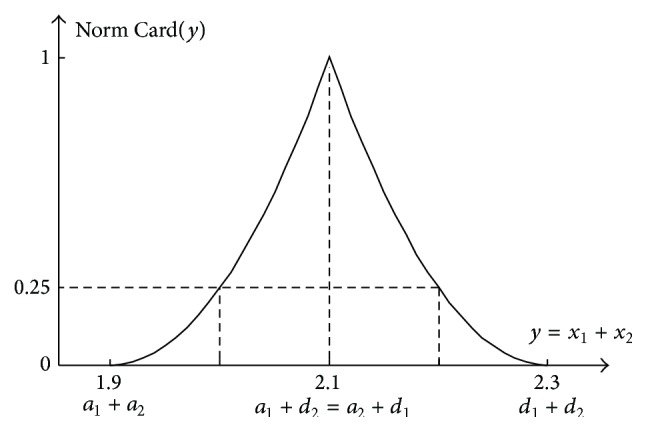
Normalised cardinality distribution Norm  Card(*y*) of the occurrence of particular values of the sum *y* = *x*_1_ + *x*_2_ being the 2D representation of the 4D addition result of two independent fuzzy numbers “about 1.0” and “about 1.1” shown in Figure 7.

The cardinality distribution of particular values of the sum *y* = *x*_1_ + *x*_2_ for the considered example is shown in Figure 10.

It should be emphasised once more that the distribution determined by formula (43) and shown in Figure 10 is not the addition result of two fuzzy numbers but only 2D information about the cardinality of particular sum values *y* = *x*_1_ + *x*_2_ derived from the full 4D addition result given by (35). The representation provided by Zadeh's extension principle (34) can also be obtained on the basis of the exact solution (35). The full 4D solution is given below. Consider

(44) y=1.9+0.2μ+0.2αx1+αx21−μαx1,αx2,μ∈0,1.

The minimal value of the sum (the left border) *y*_*L*_ for various levels of *μ* ∈ [0,1] is obtained for *α*_*x*_1__ = *α*_*x*_2__ = 0:

(45) $$\begin{array}{c} y_{L}=1.9+0.2\mu . \end{array}$$

The maximal sum value (the right border) *y*_*R*_ is obtained for *α*_*x*_1__ = *α*_*x*_2__ = 1:

(46) $$\begin{array}{c} y_{R}=2.3-0.2\mu . \end{array}$$

**Figure 11:**
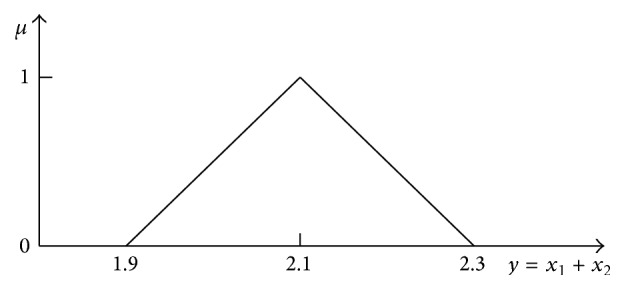
2D representation of the full 4D addition result obtained with formulas (45) and (46) corresponding to the “result” obtained with Zadeh's extension principle (34).

The 2D representation of the addition result of two fuzzy numbers “about 1.0” and “about 1.1” obtained with formulae (45) and (46) is shown in Figure 11.

The 2D representation obtained according to Zadeh's extension principle (34) informs only about the maximal spread of the full solution granule on particular *μ*-levels (Figure 9). Geometrically, the distribution from Figure 11 corresponds to the cross section going from the corner *y* = *a*_1_ + *a*_2_ = 1.9 to the corner *y* = *d*_1_ + *d*_2_ = 2.3 through the peak of the membership function in Figure 9.

## 4. Addition of Two Fuzzy Numbers with Taking into Account the Order Relation

Let us analyse an example of a fuzzy number addition similar to that in Section 3, but with small difference, with the additional knowledge that not only MFs of added numbers but also their order relation is known.

**Figure 12:**
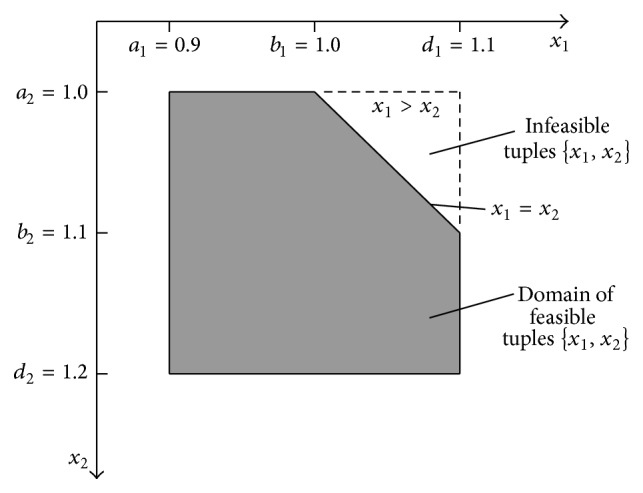
Domain of feasible tuples of weight values {*x*_1_, *x*_2_} which can take part in adding of fuzzy numbers *x*_1_ and *x*_2_.

Thus, there are two stones S1 and S2 which had been weighed on spring scales with maximal error equal to 0.1 kg. Therefore, weights *x*_1_ and *x*_2_ of stones are uncertain and can be expressed in the form of fuzzy numbers: *X*_1_ is [0.9,1.0,1.1] and *X*_2_ is [1.0,1.1,1.2]. MFs of uncertain weights are shown in Figure 7. After weighting the stones on the spring scales, their weights were compared on balance scales. The scales showed that the weight *x*_2_, though uncertain, is greater than *x*_1_. Therefore, the weights order *x*_2_ > *x*_1_ is known and can be taken into account in the weights' adding. The knowledge of the order relation changes (constraints) the domain of possible weight tuples {*x*_1_, *x*_2_} in comparison with the example from Section 3. Now less tuples will be feasible (Figure 12).

In the considered example, uncertain weights *x*_1_ and *x*_2_ are expressed by the same horizontal MFs as given by formulas (32) and (33). However, the addition result *y* is determined by more constraints (formula (47)) as in the case without the weights' order relation. Consider

(47) x1a1,d1,x2a2,d2,x1<x2,yx1+x2=a1+a2+μb1+b2−a1−a2+αx1d1−a1+αx2d2−a21−μ,y1.9+0.2μ+0.2αx1+αx21−μ,αx1,αx2,μ∈0,1.

**Figure 13:**
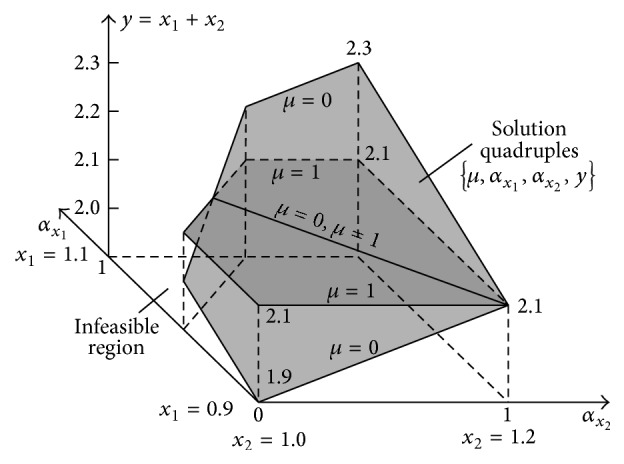
3D projection of the 4D granule of the addition result of two fuzzy numbers “about 1.0” and “about 1.1” onto the space *X*_1_ × *X*_2_ × *Y*.

Equations (47) determine the full and precise addition result *y* = *f*(*α*_*x*_1__, *α*_*x*_2__, *μ*) that has a form of the 4D information granule. This granule cannot be visualised. However, we can visualise its 3D projection onto the space *X*_1_ × *X*_2_ × *Y* that is shown in Figure 13.

**Figure 14:**
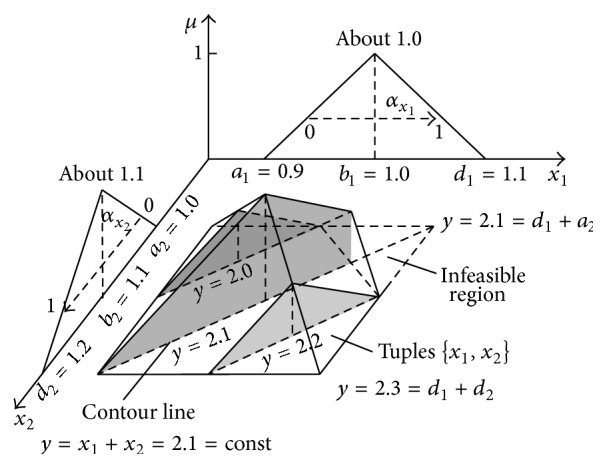
Projection of the full 4D result granule of the addition of two fuzzy numbers “about 1.0” and “about 1.1” with taking into account the order relation *x*_2_ > *x*_1_ onto the 3D space *X*_1_ × *X*_2_ × *μ*.

A comparison of addition result granules obtained without (Figure 8) and with (Figure 13) taking into account the order relation *x*_2_ > *x*_1_ shows that it considerably changes the granules shape.  Figure 14 shows other projection of the full 4D result granule onto the space *X*_1_ × *X*_2_ × *μ*. Values of the sum *y* = *x*_1_ + *x*_2_ are shown in this figure in a form of contour lines of constant *y* values.

The granule of the addition result from Figure 14 can be compared to the result granule of addition without the order relation *x*_2_ > *x*_1_ shown in Figure 9. The comparison shows that the relation *x*_2_ > *x*_1_ considerably decreases the domain of possible solutions. At the same time, we can observe decrease of cardinalities of particular *y* values for *y* ∈ [2.0,2.2]. Now, formulas for cardinalities take new forms: (50)–(56). Consider

(48) $$\begin{array}{c} \text{Max Abs Card}=\frac{1}{2}\cdot \frac{7}{8}\cdot \sqrt{{\left(d_{2}-a_{2}\right)}^{2}+{\left(d_{1}-a_{1}\right)}^{2}}. \end{array}$$

For *y* ∈ [*a*_1_ + *a*_2_, *a*_1_ + *b*_2_] = [1.9,2.0],

(49) Abs  Cardy87y−a1+a2d1−a12·Max  Abs  Card,

(50) Norm  Cardy87y−a1+a2d1−a12=87y−1.90.22.

For *y* ∈ [*a*_1_ + *b*_2_, *a*_1_ + *d*_2_] = [2.0,2.1],

(51) Abs  Cardy=87y−a1+a2d1−a12−18y−a1+b2d1−b12·Max  Abs  Card,

(52) Norm  Cardy=87y−a1+a2d1−a12−18y−a1+b2d1−b12=87y−1.90.22−18y−2.00.12.

For *y* ∈ [*a*_1_ + *d*_2_, *b*_1_ + *d*_2_] = [2.1,2.2],

(53) Abs  Cardy=87d1+d2−yd2−a22−18b1+d2−yb1−a12·Max  Abs  Card,

(54) Norm  Cardy=87d1+d2−yd2−a22−18b1+d2−yb1−a12=872.3−y0.22−182.2−y0.12.

For *y* ∈ [*b*_1_ + *d*_2_, *d*_1_ + *d*_2_] = [2.2,2.3],

(55) Abs  Cardy87d1+d2−yd1−a12·Max  Abs  Card,

(56) Norm  Cardy87d1+d2−yd1−a12=872.3−y0.22.

**Figure 15:**
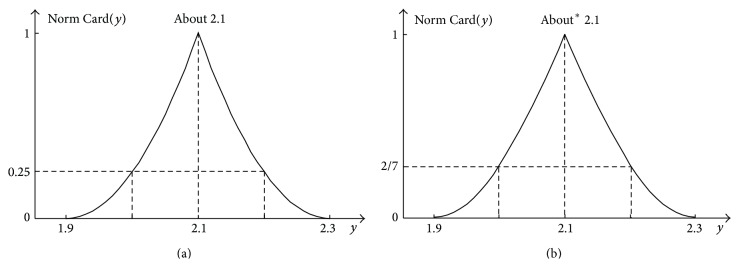
2D representation of the full 4D result in a form of the cardinality distribution for the case of the addition of two independent fuzzy numbers (a) and for the case of the addition of two fuzzy numbers constrained by the order relation *x*_2_ > *x*_1_ (b).

Figures 15(a) and 15(b) show the comparison of 2D representations of full 4D addition results in a form of cardinality distributions Norm  Card(*y*) determined by (50)–(56).

**Figure 16:**
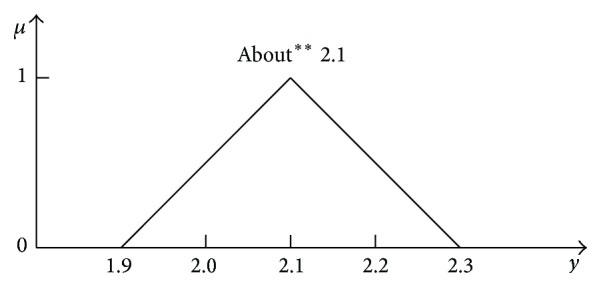
2D representation of the full 4D addition result obtained with use of the extension principle (34) both for the case of two independent numbers and dependent numbers constrained by the order relation *x*_2_ > *x*_1_.

It should be noticed that the 2D representation obtained with use of Zadeh's extension principle (34) is still identical and has the form shown in Figure 16 independently whether the addition is realised with or without taking into account the order relation *x*_2_ > *x*_1_.

The extension principle (34) does not “perceive” the domain loss (compare Figures 8 and 13) which is its important drawback. It provides even less information about the full 4D addition result than the 2D representation determined with formulas (50)–(56) and shown in Figure 15.

## 5. Addition of Two Fully Dependent Fuzzy Numbers

Correlations between variables have no significance in the case of an addition of two crisp numbers. However, in the case of intervals, fuzzy intervals, and fuzzy numbers, mutual dependencies are of great importance. Let us consider another example.

Small John did not know exactly how much money he had in his money box after a month of saving. He evaluated that the sum was about $10, at least $7, and no more than $13. This evaluation can be expressed by the fuzzy number [46, 49, 51]. His father promised to double the saved sum after 1 month. He opened the box, checked the sum, and added exactly the same sum to the box. However, father did not inform John of the sum he added. How much money does small John have in the box now?

Let us denote by *x*_1_ the uncertain sum of money which small John had in the money box at the beginning: *x*_1_ is “about 10” [46, 49, 51]. The horizontal MF of the set “about 10” can be determined on the base of formula (32):

(57) $$\begin{array}{c} x_{1}=7+3\mu +6\alpha_{x_{1}}\left(\;1-\mu \;\right),\;\mu ,\alpha_{x_{1}}\in \left[0,1\right]. \end{array}$$

Though the precise value of the sum *x*_1_ is not known for us and for John, his father knew it and added the same sum *x*_2_ to John's money box. Because *x*_1_ = *x*_2_, then from (58) we can conclude that *α*_*x*_1__ = *α*_*x*_2__. Consider

(58) x17+3μ+6αx11−μ=x2=7+3μ+6αx21−μ,αx1,αx2,μ∈0,1.

**Figure 17:**
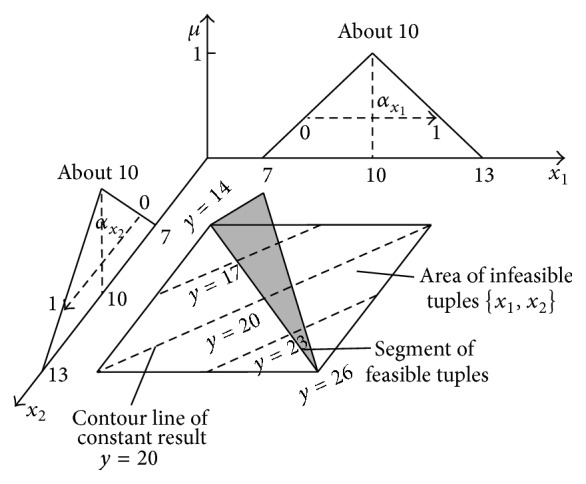
Visualization of the addition result of two fully dependent fuzzy numbers: “about 10” + “about 10” for which the condition *α*_*x*_1__ = *α*_*x*_2__ has to be satisfied.

It means that both RDM variables and also variables *x*_1_ and *x*_2_ are mutually fully coupled (correlated). The resulting sum *y* = *x*_1_ + *x*_2_ can be expressed by the following formula:

(59) y=x1+x2=14+6μ+61−μαx1+αx2,αx1=αx2  αx1,αx2,μ∈0,1,

and it is visualised in Figure 17.

As Figure 17 shows, the addition result “about 20” is in this case the flat and not 3D information granule as in the case of the addition of fully independent fuzzy numbers; see Figure 9. As before, the flat 2D representation in the form of the horizontal MF *y* = *f*(*μ*) can be determined on the basis of exact solution (59). This function can be characterized with left and right border functions *y*_*L*_(*μ*) and *y*_*R*_(*μ*). Both functions can be determined from the full result:

(60) $$\begin{array}{c} y=14+6\mu +12\alpha_{x_{1}}\left(\;1-\mu \;\right). \end{array}$$

The left, minimal function border is obtained for minimal values of RDM variables *α*_*x*_1__ = *α*_*x*_2__ = 0. Consider

(61) $$\begin{array}{c} y_{L}\left(\;\mu \;\right)=14+6\mu . \end{array}$$

The right border of the 2D representation, the border of maximal *y* values, is obtained for maximal values of RDM variables *α*_*x*_1__ = *α*_*x*_2__ = 1. Consider

(62) $$\begin{array}{c} y_{R}\left(\;\mu \;\right)=26-6\mu . \end{array}$$

**Figure 18:**
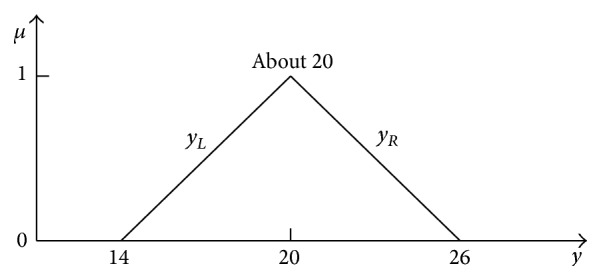
2D representation of the full 4D result (59) *y* = *f*(*μ*, *α*_*x*_1__, *α*_*x*_2__) of the addition of two fully dependent fuzzy numbers “about 10” + “about 10” satisfying the condition *α*_*x*_1__ = *α*_*x*_2__.

2D representation of the full 4D addition result (59) is shown in Figure 18.

## 6. Solving Additive Fuzzy Equation: *A* + *X* = *C*

As it was mentioned in Section 1, solving equations with one unknown *x* makes great difficulties even for the interval arithmetic which is considerably simpler than the fuzzy arithmetic. It will be shown in this section that solving fuzzy equations with use of horizontal MFs is possible and not difficult. Let us consider the last example.

Corn harvested from field 1 was weighed on the scales that have maximal error equal to 1 ton. The scales indicated 5 tons. Thus, the true weight *a* of the corn can be expressed in a form of the fuzzy number *A* = [4,5, 6]. Corn harvested from field 2 was not weighed on the field. Therefore, its true weight *x* was not known even approximately. Both crops were brought to a warehouse. There, they were mixed and weighed together on 3 scales that also have the maximal error equal to 1 ton. The scales indicated 15 tons. Thus, the sum *c* of crops can be expressed in a form of the fuzzy number *C* = [14,15,16]. How large is the weight *x* of the crop from field 2? The knowledge of *a* and *x* is important, because farmers 1 and 2 should be paid fairly for their delivery.

This problem cannot be solved uniquely with *μ*-cut method based on the traditional interval arithmetic. However, it can be solved with an application of horizontal MFs (63) which can be constructed on the basis of the general formula (12). Consider

(63) A4,5,6:  a=4+μ+2αa1−μ,C14,15,16:  c=14+μ+2αc1−μ,μ,αa,αc∈0,1.

Value *c*, though known only approximately, is the result of the addition of also only approximately known values *a* and *x*. They are connected by a dependence *a* + *x* = *c*. Thus, the unknown value *x* of crop from field 2 can be calculated from the following formula:

(64) xc−a=14+μ+2αc1−μ−4+μ+2αa1−μ=10+2αc−αa1−μμ,αa,αc∈0,1.

**Figure 19:**
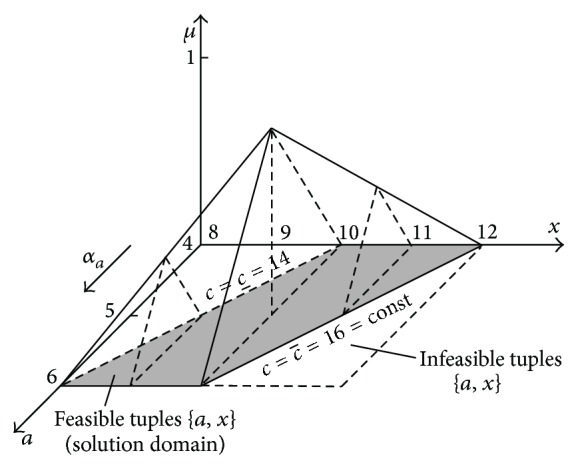
3D projection of the full 4D solution of (64) from the space *A* × *C* × *X* × *μ* onto the space *A* × *X* × *μ*.

It can be easily noticed that formula (64) is four-dimensional: *x* = *f*(*μ*, *α*_*a*_, *α*_*c*_). It cannot be expressed exactly in the 2D space, which is suggested by the extension principle and various versions of the fuzzy arithmetic. However, it can be visualised in a form of the 3D projection on subspaces, for example, on the subspace *A* × *X* × *μ* (variable *α*_*a*_ is equivalent to variable *a* because of the transformation *a* = 4 + 2*α*_*a*_) (Figure 19).

**Figure 20:**
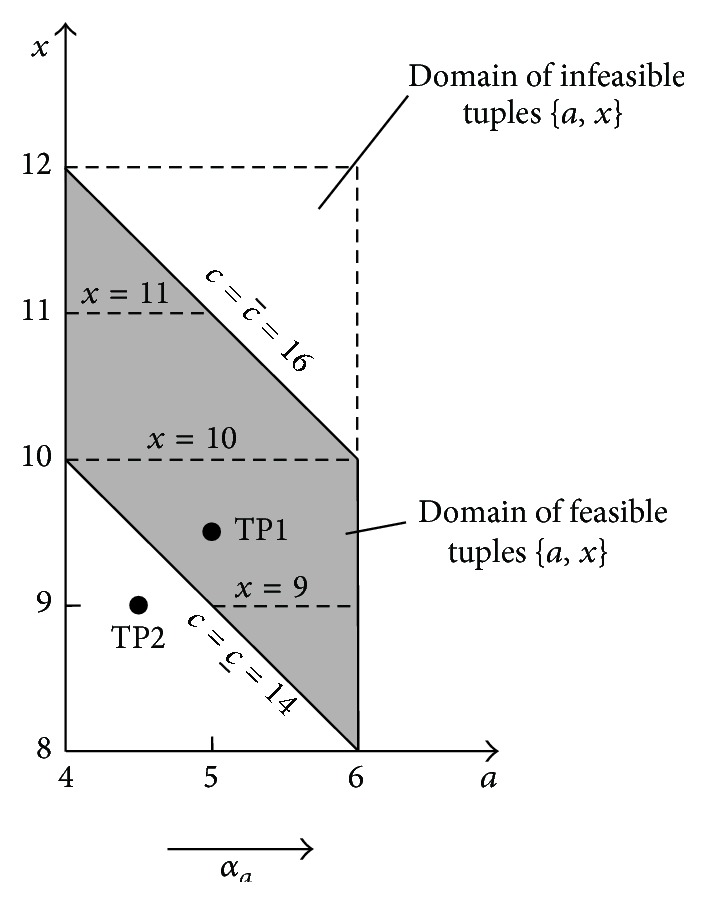
Support of the 3D membership function of the solution from Figure 19. TP1 = (5,9.5) and TP2 = (4.5,9) are examples of test points that can be used for the correctness testing of obtained solutions.

Figure 19 shows the support of the membership function of solution (64) being the *μ*-cut of this function on the level *μ* = 0. It can be noticed that this support does not have a shape of a rectangle but of a parallelogram, which is shown additionally in Figure 20.

Figure 20 shows that the solution of the fuzzy equation *A* + *X* = *C* is not 1-dimensional one and therefore it is not possible to express it in a form of the interval $\left[\underline{x},\bar{x}\right]$ which is usually done by these versions of fuzzy arithmetics that are based on the classical IA. Moore's IA provides us with two possible solutions: (65) and (66). The first result (65) is obtained if the equation *A* + *X* = *C* is solved for the cut *μ* = 0. Consider

(65) A+X=C,4,6+x_,x¯=14,16,4+x_=14,x_=10,6+x¯=16,x¯=10,x_,x¯=10,10.

Solution (65) represents only one of many possible solution subsets: it is the subset containing tuples {(*a*, *x*)∣*x* = 10}. However, apart from this subset, there is also an infinitely large set of solution tuples {*a*, *x*} which satisfy the interval equation *A* + *X* = *C*. Example of such tuple is the test point TP1 (*a*, *x*) = (5.0,9.5) in Figure 20, because, for *a* = 5 ∈ [4,6], the sum *a* + *x* = *c* = 5 + 9.5 = 14.5 ∈ [14,16].

The second possible solution of the equation *A* + *X* = *C* for *μ* = 0 gives formula (66). Consider

(66) A+XC,4,6+x_,x¯14,16,x_,x¯14,16−4,6=8,12.

One can easily convince himself that this result is not the precise solution of the considered equation. It is only information about the greatest span [8,12] of the precise solution (64), which has a form of a parallelogram (Figure 20). As can be seen in Figure 20, for the interval $\left[\underline{x},\bar{x}]=[8,12\right]$ , we can create an infinite number of tuples {*a*, *x*} which are not solutions of the considered equation. For example, the tuple (4.5,9) corresponding to the test point TP2 has coordinates *a* = 4.5 ∈ [4,6] and $x=9\in [8,12]=[\underline{x},\bar{x}]$ . Above-mentioned examples show that only multidimensional approach to the fuzzy and interval arithmetic can give correct problem solutions. It should be noticed that the maximal solution span [8,12] provided by Moore's arithmetic can be easily obtained from the multidimensional solution (64) of equation *A* + *X* = *C*. If we are interested in the solution span on the cut level *μ* = 0, then this value of *μ* can be inserted into (64) being 4D solution and, as a result, we obtain

(67) $$\begin{array}{c} x=c-a=10+2\left(\;\alpha_{c}-\alpha_{a}\;\right),\;\alpha_{a},\alpha_{c}\in \left[0,1\right]. \end{array}$$

**Figure 21:**
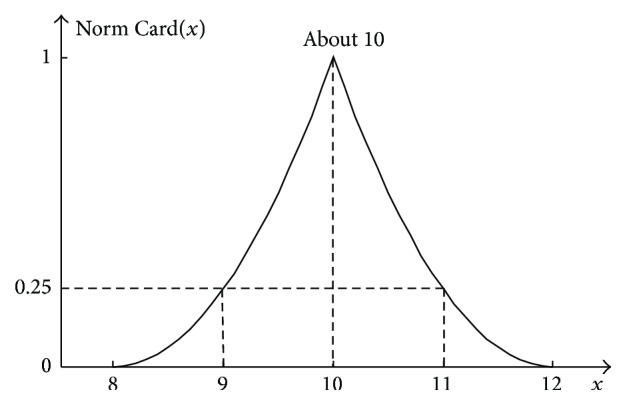
2D distribution of the full 4D solution of the fuzzy equation *A* + *X* = *C* in a form of the cardinality distribution of particular *x* values, *x* ∈ [8,12].

A simple analysis of this equation gives the conclusion that the minimal value $\underline{x}$ occurs for *α*_*a*_ = 1 and *α*_*c*_ = 0. This value equals $\underline{x}=8$ . The maximal value $\bar{x}$ occurs for *α*_*a*_ = 0 and *α*_*c*_ = 1. It is equal to 12. Hence, the maximal solution span $\left[\underline{x},\bar{x}]=[8,12\right]$ .  Figure 21 shows 2D representation of the full 4D solution of the equation *A* + *X* = *C* in a form of the cardinality distribution of particular *x* values.

## 7. Conclusions

The paper presents the new concept of horizontal MFs which were used for the addition as the exemplary operation on fuzzy numbers. The application of such functions in combination with the RDM arithmetic enables multidimensional approach to operations on fuzzy numbers and thereby it allows for removing some drawbacks and weaknesses of the fuzzy arithmetic. The use of horizontal MFs considerably facilitates calculations because now uncertain values can be inserted directly into equations without using the extension principle.

Additionally, the RDM arithmetic enables taking into account correlations occurring between arguments of mathematical operations which is not possible using one-dimensional approach offered by classic FA methods.
